# Patient perceptions of prosthodontic management options for partially dentate older adults: a qualitative study

**DOI:** 10.1186/s12903-026-08379-3

**Published:** 2026-04-23

**Authors:** Sarah Meaney, Cristiane DaMata, Finbarr Allen, Francis Burke, Martina Hayes, Caoimhe Quail, Ross Welsh, Ciaran Moore, Gerry McKenna

**Affiliations:** 1 Cork University Dental School and Hospital, University College Cork, Cork, Ireland; 2 National Perinatal Epidemiology Centre, University College Cork, Cork, Ireland; 3 Royal School of Dentistry, Belfast, Northern Ireland; 4 Dublin Dental School, Trinity College Dublin, Dublin, Ireland; 5 Centre for Public Health, Royal School of Dentistry, Queen’s University Belfast, Belfast, Northern Ireland; 6 Center of Excellence in Precision Medicine and Digital Health, Department of Physiology, Faculty of Dentistry, Chulalongkorn University, Bangkok, 10330 Thailand

**Keywords:** Tooth loss, Partially dentate, Older adults, Fixed prosthodontics, Removable prosthodontics, Shortened dental arch

## Abstract

**Background:**

Changes in oral epidemiology have contributed to an older population who are partially dentate in many countries worldwide. This study aims to appreciate older patients’ impressions of the loss of their natural teeth and explore their experience of functionally orientated oral rehabilitation approach.

**Methods:**

Recruitment for this retrospectively registered qualitative study involved a purposive sample of 15 older partially dentate patients. Previously, this group of patients partook in a randomised controlled clinical trial (RCT) on tooth replacement options. Patients who had received functionally orientated tooth replacements according to the Shortened Dental Arch (SDA) principles were invited to participate in a qualitative interview. Each patient received resin-bonded bridgework (RBB) to restore them to a functional dentition of 10 occluding pairs. All patients had previous experience with tooth replacement using Removable Partial Dentures (RPD). Following comprehensive interviews with participants, data was interpreted through thematic analysis.

**Results:**

This study’s findings indicated high participant satisfaction with SDA treatment. Patients noted they found resin-bonded prostheses “lightweight,” “neat,” and “fixed”, citing these as contributing factors to the ease with which they adapted to these prostheses. It was also noted that patients perceived their new prostheses to be durable and an improvement on past removable treatments received. However, the patients indicated concern that the SDA treatment provided in the clinical study would not be available to them in a primary care setting.

**Conclusions:**

This qualitative study demonstrates that partially dentate older patients were satisfied with a functionally orientated approach to oral rehabilitation.

**Trial registration:**

(Trial Registration: ISRCTN26302774: 18/10/2013).

**Supplementary Information:**

The online version contains supplementary material available at 10.1186/s12903-026-08379-3.

## Background

Oral health profiles have changed significantly across Europe over the last two decades [1]. These changes indicate an attitudinal shift in how older age groups view their oral health. This trend has been noted in the recent update to the Adult Oral Health Survey 2023^2,^ with a greater proportion of older patients in England being partially dentate rather than edentulous. This can be attributed to improved preventive programmes and changing patient attitudes, including a desire for treatments that maintain rather than replace natural teeth [3]. Although positive, improved retention of natural teeth in older people presents challenges, as older adults are typically more likely to have chronic dental diseases such as caries, tooth wear and periodontitis [4–6]. Also, older individuals from more deprived socio-economic areas have been shown to have fewer natural teeth [2]. Hence, it is essential that cost-effectiveness and patient acceptability are considered when evaluating prosthodontic management options for such patients’ dentitions.

The factors influencing treatment choices for partially dentate older patients are poorly understood. This is particularly true of treatment choices for replacing missing teeth. Removable partial dentures (RPDs) are commonly used to replace missing teeth. However, many patients are non-compliant with wearing RPDs, particularly when they are used to replace posterior teeth [7, 8]. RPDs can also pose challenges during maintenance and can complicate the patient’s oral hygiene. Alternative options to RPDs are available. One such alternative is the Shortened Dental Arch (SDA) concept, a functionally oriented treatment that focuses on preserving a reduced but functional dentition and avoids replacing missing posterior teeth with removable prostheses.

The SDA concept has been successfully implemented in older patients by focusing treatment on preserving anterior teeth, which are easier for patients to maintain [9, 10]. A natural SDA can be achieved in patients with 20 natural teeth. However, the number of patients who satisfy this criterion is small. It is more likely that patients in the older age group will require restoration to an SDA. This restoration will typically involve a range of fixed prosthodontic options, including dental implants and resin-bonded bridgework (RBB) [11].

RBB has been shown to be an effective and simple method for replacing missing teeth, providing patients with an SDA [10]. Within the randomised control trial in which this study is nested, selected RPD and RBB provision was compared. RBB was selected as the modality because it is minimally invasive and, unlike dental implants, does not require an invasive surgical procedure, which could be perceived negatively by older patients [12]. The success rates of RBBs are also favourable, with survival of up to 87.7% reported after 5 years [13].

SDA is a technique widely recognised by clinicians; recent literature supports the view that there is no association between this functional approach and the causation of pathology [14]. Certainly, studies have noted that there is no discernible difference in the impact on the periodontium when using such an approach compared with restoration with RPD or other restorative techniques [15, 16]. Thus, SDA is indicated as a reasonable alternative to conventional techniques when cost-effectiveness and medical complexity are considered [17] owing to its lower maintenance burden.

The current literature in health-related research clearly demonstrates the value of adopting qualitative approaches to support practitioners in gaining insight into patients’ motivations and perspectives [18, 19]. Such knowledge is instrumental in providing clinicians with sufficient information for patient-centred treatment planning and to play a critical role in the decision-making process for patients.

As previously mentioned, the population’s age profile in Europe is increasing. Many more people require complex health care. There is already evidence of inequality of access to preventative oral healthcare for older people [20]. Adults over fifty are finding the cost of oral healthcare a barrier to accessing these services [21]. It is vital that new evidence-based approaches to oral health care are developed, as these will not only produce better clinical outcomes for older patients but also make this care more cost-effective [22] whilst ensuring that it remains acceptable to this patient group.

This study aims to gather information and develop a better understanding of older people’s attitudes towards tooth loss and their experiences of a functionally oriented approach to oral rehabilitation. Considering the opinions and motivations of this growing patient group is crucial for clinicians to ensure that treatment decisions centre on the informed patient.

## Methods

This retrospectively registered qualitative study was nested within a randomised controlled clinical trial (RCT) (Trial registration: ISRCTN26302774: 18/10/2013). The in-depth methodology of the RCT has been delineated in numerous previous publications, and so a brief summary is provided here [23–25]. This RCT adhered to CONSORT guidelines. The inclusion criteria for patients were those who were 65 years or older and seeking replacement of missing natural teeth. Full ethical approval was granted for the study (ref: ECM 5 (9) 05/02/08). Patients were randomly allocated to two treatment groups: the RPD and SDA groups. Each patient from the RPD group had all missing natural teeth replaced with RPDs, whilst each patient from the SDA group was restored to a functional occlusion of 10 occluding pairs of natural and replacement teeth using RBB throughout the arch [24]. The collection of qualitative data was conducted as a separate study; however, the patients were recruited from the original randomized controlled trial (RCT). At the inception of the RCT, a qualitative component was not considered.

Recruitment for this qualitative study involved a purposive sample of 15 older (65 years and older) partially dentate patients who received functionally oriented treatment (SDA group) as part of the RCT (a group of 45 patients [23]), all of these patients before treatment would have had a minimum of six remaining natural teeth in both arches of good prognosis, were able to accept routine dental care in a dental chair, could communicate in English and had no medical conditions which precluded routine dental treatment [23]. A summary of their demographics is provided in Table 1.

**Table 1:** Patient demographics of participants in a qualitative study of patient perceptions of prosthodontic management options for partially dentate older adults

Participant Number	Gender	Age (Years)	Number of remaining natural teeth (*n*)
1	Female	68	16
2	Female	71	17
3	Male	72	15
4	Male	69	18
5	Male	66	16
6	Female	70	15
7	Female	74	17
8	Male	77	17
9	Female	68	16
10	Male	70	16
11	Female	72	14
12	Female	74	17
13	Female	75	17
14	Male	70	16
15	Female	68	14

The qualitative data collection was completed following the conclusion of the RCT. Qualitative data were collected from February 2017 to November 2017, and the data were then transcribed and analysed in 2018.

Patients were provided with a written information sheet at their 3-year review appointment explaining the purpose of the qualitative study. If patients indicated that they were interested in participating in the qualitative aspect of the research study an interview was arranged at a time of their convenience. All patients in the purposive sample agreed to participate in the study. All of the patients recruited had previous experience with RPDs prior to receiving SDA treatment.

Interviews were undertaken by a social researcher experienced in qualitative methods and with an educational background that includes a Master’s degree in applied social research. This social researcher was not involved in any aspect of their clinical management. None of the participants were known to the researcher. Before the interview commenced, the researcher reiterated that participation was voluntary whereby they could decline to partake in the study at any point. All participants in the study provided written informed consent and gave their time freely.

Interviews with nine females and six males were conducted in a private room in Cork University Dental School and Hospital. The surroundings of the dental hospital outpatient setting were familiar to the participants as they attended there for treatment. All the interviews were conducted in a quiet room, a short distance from this clinical setting. Semi-structured interviews were conducted using a semi-structured topic guide and a neutral approach. As the interviews were semi-structured, participants were encouraged to discuss additional areas they considered pertinent, thus allowing the thorough exploration of the participant’s experience of oral rehabilitation. The topic guide included the following areas of exploration;

- previous dental history and decision-making related to treatment;
- treatment and management options, including tooth replacement options;
- adaption to tooth replacement treatment,
- function of dental prostheses provided;
- expectations for future dental treatment and management.

The interviews were digitally recorded and transcribed verbatim.

Data collection and thematic analysis, as outlined by Braun and Clarke, proceeded concurrently [26]. As with all qualitative research approaches, it is acknowledged that analysis is complicated by the researcher’s own perspective, and therefore, the analysis of these data from a different perspective may produce different results [27]. The analysis and interpretation of the data was carried out by SM using NVivo 11 software (QSR International Pty Ltd., Doncaster, Australia). The analyses were then presented to co-authors for review and are presented here. These analyses included familiarisation of the data and the identification of the preliminary codes. Similar individual codes were re-organised into categories. The final phase of analysis involved the re-organisation of categories in order to develop themes that are represented across the whole dataset.

## Results

The research team discussed the interviews and the emerging themes throughout the data collection phase. Data saturation was reached following 15 in-depth interviews with participants. The data were categorised into four main themes, namely: negative perceptions of previous dental care; experience with RPDs to replace missing teeth; experience of SDA treatment; and important factors in patient decision-making.

### Negative perceptions of previous dental care

Patients discussed their previous experience with the dental treatment which they had received over their lifetime from General Dental Practitioners (GDPs). All of the participants expressed negative perceptions of their previous dental treatment, particularly when they began to lose their teeth decades previously. Patients discussed that the treatment offered to them previously was very much centred around the extraction of teeth, with no mention of preventative or operative care provided by their dentist to help them retain their natural teeth.

*“Every time I got a tooth ache*,* I got the tooth pulled. I never got it filled. Anytime any bit of toothache*,* next morning straight to the dentist to the pull the tooth… that was our system and that is the way it went on and we [generation] never got our teeth filled.”* *(Participant 3; Male).*

*“Long ago*,* not now*,* but they [dentists] were very good at taking them [teeth] out*,* the easiest thing was take them [teeth] out and that is how I lost a few [teeth]. There was nothing else*,* you didn’t fill them. I didn’t think of it at the time*,* they [teeth]could have been paining me [painful] and to get rid of the pain was good…and you don’t realise at the time how important it is*,* and he [dentist] just took them out.”* *(Participant 1; Female)*.

Many of the participants spoke about their reluctance to attend a GDP today due to their concerns about extractions being the only treatment available to them for any remaining natural teeth. The patient group discussed that many of them were not regular dental attenders prior to entering the clinical study and would only attend for acute issues rather than regular check-ups.

### Experience with RPDs to replace missing teeth

All of the patients interviewed discussed their previous experiences with RPDs. Many discussed the limitations of RPDs, describing feelings of “discomfort,” feeling “bulky,” and having a “foreign object” in their mouths. A number of patients discussed specific functional limitations they experienced with their RPDs, including speaking, eating, and laughing, with an associated lack of confidence in social interactions.

*“I had a metal plate (RPD) with two teeth on it*,* and I had that for 25 or 30 years and I lost it*,* so I had another one put in and it was not at all as satisfactory. It was a big monstrosity in my mouth*,* it wasn’t neat*,* there was a lot of*,* the roof of my mouth filled up with metal and that was the main problem with it…* *(Participant 10; Male)*.

*“I know about false teeth. I talked to my friends about them and how they couldn’t eat and how they slipped [laughing] and that put me off false teeth forever”* *(Participant 9; Female).*

Patients also discussed the high maintenance requirements of RPDs with a number discussing a large number of initial appointments for denture adjustments following construction of a new RPD and the ongoing need for repairs.

*“My dentures would break*,* I have a heavy bite when I eat and I was always breaking them and I would be going to the dental mechanic to fix them.”* *(Participant 3; Male).*

### Experience of SDA treatment

Patients’ overall satisfaction with the SDA treatment was a consistent finding. Many of the patients referred to how they adapted to the resin-bonded bridgework as part of SDA treatment with relative ease as it was “lightweight” and “neat” in their mouth. The fixed aspect of the SDA treatment approach was considered one of the most positive aspects for many of the participants, and they reported that after a short period of adjustment, the fixed bridgework felt like it was almost part of their mouth. Participants who received SDA treatment consistently discussed how they would be reluctant to return to RPD treatment, which would include regular removal of a denture. In fact, a number of patients suggested that they would simply accept unrestored missing teeth rather than consider an RPD again.

*“They did an absolutely superb job putting the bridge in there*,* it took a lot of effort*,* but it got there and I am absolutely over the moon with it …it’s [the SDA treatment] tidier*,* neater more streamlined*,* it is just like my natural teeth*,* I have no plate in my mouth*,* it’s perfect”* *(Participant 10; Male).*

*“I found this treatment [SDA treatment] fantastic”* *(Participant 9; Female).*

Patients’ attributed their high levels of satisfaction with the SDA treatment to how treatment options had improved over the years since they first sought dental treatment for the loss of their teeth. Participants spoke of how improvements in technology and materials have led to improved treatments, which they perceive to be durable and long-lasting. They recounted how such treatment options were not available to them when they were initially seeking treatment, as the only option presented to them was an RPD.

Following the fitting of the RBB, the patients spoke of improved confidence in their daily lives, and they linked these feelings to the improved oral function and aesthetics achieved from the bridgework. Participants spoke of the renewed sense of security they now felt when undertaking daily tasks, primarily related to eating and chewing. The participants reported improved confidence in public situations, as they no longer had concerns that something may happen to their teeth while eating or speaking that might lead to embarrassment.

*“I can eat everything now*,* I like nuts*,* I like nuts a lot and I eat those now*,* cashews and pistachios. But steak before [the SDA treatment] you would only ever half chew it and that wasn’t good you know if it was half chewed and you were trying to swallow it. So*,* now I can eat everything”* *(Participant 7; Female)*.

### Important factors in patient decision-making

The patient group voiced their concerns that the SDA treatment which they had been provided with as part of the RCT would not be available to them if they attended their dentist in primary care. Some of these patients believed that they would not have the right to such prosthetics under state-funded care and that the costs involved would be prohibitive. These patients reported that, as they believed the treatment would be very expensive, they would have difficulty justifying spending their money on such treatment. These patients reported that if they were younger, they would give more consideration to spending money on this sort of treatment.

*“Our [financial] circumstances wouldn’t allow me to spend that money on myself.”* (Participant 12; Female).

The dynamic between the oral healthcare professionals and these older patients had an impact on the care which they sought and the satisfaction they received from their treatment. Some of the group had negative opinions about GDPs where proposed treatment options were not discussed but expected to be accepted. Interestingly, despite the reliance on clinicians to make well-informed decisions on their behalf based on expert opinion (an opinion which patients believed was of more importance than their own experience and expectations for this care), their avoidance of attending GDPs for fear of further tooth extractions was illustrative of their own personal preferences for oral healthcare.

Participants valued the time spent with them to talk through treatment as part of the clinical study. They felt fully informed but they also felt that they were being listened to if they had questions. The patients valued the follow-up care that they were receiving as part of the study including regular reviews. The patients consistently mentioned the importance of building a trusting relationship between clinician and patient.

*“What I like about him [dentist in the dental hospital] very much is that he will explain everything to you*,* while before it was just open your mouth*,* you need that done and bang the needle is in you know. And I find everything much more gentle which is great*,* especially if you are a nervous person like I am…****” ****(Participant 12; Female).*

## Discussion

This study is the first to capture partially dentate older patients’ options on the application of functionally orientated tooth replacement using a qualitative methodology. This study clearly demonstrated patients’ positive perceptions of SDA treatment compared to previous RPDs. Patients felt that SDA treatment with RBB used to replace missing teeth had helped to improve their confidence with eating, chewing and in social situations. These results have been mirrored in other studies, albeit with data collected using different methodologies. Researchers from the University of Newcastle restored patients to an SDA in the lower arch and measured patient satisfaction in areas such as appearance and perceived masticatory ability using a questionnaire. Changes in summary satisfaction scores indicated improved satisfaction after the provision of functionally orientated treatment [28]. Another study carried out in Germany measured improvements in oral health-related quality of life (OHRQoL) for patients restored to an SDA using the OHIP-49 questionnaire. Although the treatment interventions differed from this study, statistically significant improvements in OHRQoL were also demonstrated [29]. Data from this RCT has also demonstrated consistent improvements in OHRQoL for patients restored to an SDA using the OHIP-14 questionnaire at both one and 2-year follow-up [23, 30]. These scores have demonstrated improvement in functional limitation, which has been further corroborated using objective masticatory function tests [31]. All of the patients who participated in the qualitative study had previous experience of RPDs and many expressed a reluctance to consider a removable prosthesis again, indeed a number would simply accept missing teeth rather than reconsider this treatment option which does come at a biological price including increased incidence of caries [32–35].

Other studies have been conducted within Europe and the rest of the world on the SDA concept, such as one within South Africa, which supports the acceptability of SDA and emphasises the importance of education on this topic [36]. Further, an observational cohort study conducted in the Netherlands notes longevity of such an approach, and by contrast, the provision of free-end RPDs is not advised owing to less favourable clinical outcomes over time [37].

In our study, patients exhibited high satisfaction with being restored to an SDA whilst in the wider literature, its acceptability remains debated. A Manchester study found that their patient group did not consider an SDA approach to be more useful than prosthetic replacement of a conventional arch [38]. This suggests that, while our results show SDA can be acceptable and effective, it’s important to recognise individual patient preferences, as highlighted by Manchester’s findings and thus consider other prosthetic interventions such as RPDs.

More recent systematic evidence from 2017 also suggests that SDA and conventional prosthetic strategies, such as removable partial dentures, yield similar oral health-related quality-of-life outcomes at annual follow-up [39]. This nuance challenges the notion that complete arch replacement is always preferable from an OHRQoL standpoint, but also underscores that SDA is not invariably superior across patient populations.

This study has limitations, as bias is introduced by the inherent differences in the treatments provided to the interviewed patients, including, but not limited to, differences in setting and available resources. The RPDs provided were generally completed in a dental practice, whereas the SDA treatment was supplied at the dental hospital. Other forms of bias include a small sample size and selection bias due to the recruitment method, with all participants recruited from a single institution in a single country. Furthermore, all participants reported negative experiences with RPDs previously provided, without the clinical reasons for these experiences; this does not provide a full clinical picture and may have biased attitudes towards SDA treatment, particularly as patients who had positive experiences with RPDs were not included. These factors could be addressed through further comparative studies conducted within the same setting (GDP or hospital), with a larger cohort of patients, and by collecting data across multiple centres, thus building on this study, reducing bias, and improving external validity.

In this study, this patient group appeared to have mainly negative perceptions about the treatment available to them in primary care. They expressed concerns about the availability of SDA treatment outside the study environment, a concern supported by previous evidence of its underutilisation [40]. Previous studies have consistently concluded that whilst dentists understand the benefits of functionally orientated tooth replacement, they fail to utilise it widely [41–43]. Although practitioners do recognise the limitations of RPDs in restoring a conventional arch [44]. Reasons for these behaviours could relate to training issues, including a lack of awareness and the remuneration system in which dentists work. There is also evidence to indicate that older patients encounter barriers created by the dental profession when attempting to access oral care [45]. Many practitioners reported that the treatment of older patients was time-consuming and lacking in suitable remuneration [46, 47]. However, other clinicians have indicated that they do not feel properly trained or competent to provide geriatric oral healthcare [48]. Unfortunately, this corresponds with the negative opinions expressed by the older patients within this study.

SDA research in Finland has led to the production of a guideline document [49], and the translation of this concept in this manner is important to reach appropriate stakeholders, as highlighted by Khan [50]. Further qualitative research in this area would support understanding the barriers to the provision of this functionally oriented treatment strategy in general dental practice in Europe and thus inform potential changes that could be implemented to aid the dissemination of this technique.

## Conclusion

This qualitative study further evidenced the advantages of functionally orientated treatment for older adults.

The interviews highlighted the negative experiences and perceptions related to tooth loss among this group of patients, particularly among those who felt that options for retaining teeth were not discussed thoroughly enough in the past. Thus, tooth replacement options should be kept at the forefront of a clinician’s mind, and when creating a tailored treatment plan for each patient, the patient must be involved in an open and transparent discussion about these.

RBB offers a promising treatment alternative to RPDs, particularly for partially reduced dentitions with (almost) sound remaining teeth.

This study shows that RBB is well-accepted by patients and relatively easy to place. Evidence also suggests that maintenance of a functionally orientated dentition is more achievable for the patient and ultimately more cost-effective [51]. This contradicts the patients’ views that SDA treatment would be prohibitively expensive in primary care. These factors, in combination, should encourage clinicians and policymakers to utilise this treatment concept more widely in appropriate cases.

## Supplementary Information

Supplementary Material 1.

## Data Availability

All data generated or analysed during this study are included in this published article.

## Abbreviations

SDA: Shortened Dental Arch
RCT: Randomised Controlled Clinical Trial
RBB: Resin Bonded Bridgework
RPD: Removable Partial Dentures
GDPs: General Dental Practitioners
OHRQoL: Oral Health-related Quality of Life
OHIP: Oral Health Impact Profile
