# Supplementary material for: Patient perceptions of prosthodontic management options for partially dentate older adults: a qualitative study
Source: BMC Oral Health. 2026 Apr 23;26:755. doi: 10.1186/s12903-026-08379-3 (PMC13122978; doi:10.1186/s12903-026-08379-3)
Supplement: Supplementary file 1 — Supplementary Material 1. [file 12903_2026_8379_MOESM1_ESM.docx]

# **Semi-structured Interviews**

# **Topic Guide**

# Previous dental history and decision-making related to treatment;

# Treatment and management options, including tooth replacement options;

# Adaption to tooth replacement treatment,

# Function of dental prostheses provided;

# Expectations for future dental treatment and management.
